# Association of Sociodemographic Factors, Smoking-Related Beliefs, and Smoking Restrictions With Intention to Quit Smoking in Korean Adults: Findings From the ITC Korea Survey

**DOI:** 10.2188/jea.JE20110026

**Published:** 2012-01-05

**Authors:** Seung-Kwon Myung, Hong Gwan Seo, Yoo-Seock Cheong, Sohee Park, Wonkyong B Lee, Geoffrey T Fong

**Affiliations:** 1 Smoking Cessation Clinic and Center for Cancer Prevention and Detection, Hospital, National Cancer Center, Goyang, Republic of Korea; 2 Cancer Epidemiology Branch, Research Institute, National Cancer Center, Goyang, Republic of Korea; 3 Department of Family Medicine, Dankook University College of Medicine, Cheonan, Republic of Korea; 4 Cancer Biostatistics Branch, Research Institute, National Cancer Center, Goyang, Republic of Korea; 5 DAN Program in Management and Organizational Studies, University of Western Ontario, London, Canada; 6 Department of Psychology, University of Waterloo, Waterloo, Canada; 7 Ontario Institute for Cancer Research, Toronto, Canada

**Keywords:** intention to quit, sociodemographic factors, smoking-related beliefs, smoking restrictions, smokers

## Abstract

**Background:**

Few studies have reported the factors associated with intention to quit smoking among Korean adult smokers. This study aimed to examine sociodemographic characteristics, smoking-related beliefs, and smoking-restriction variables associated with intention to quit smoking among Korean adult smokers.

**Methods:**

We used data from the International Tobacco Control Korea Survey, which was conducted from November through December 2005 by using random-digit dialing and computer-assisted telephone interviewing of male and female smokers aged 19 years or older in 16 metropolitan areas and provinces of Korea. We performed univariate analysis and multiple logistic regression analysis to identify predictors of intention to quit.

**Results:**

A total of 995 respondents were included in the final analysis. Of those, 74.9% (*n* = 745) intended to quit smoking. In univariate analyses, smokers with an intention to quit were younger, smoked fewer cigarettes per day, had a higher annual income, were more educated, were more likely to have a religious affiliation, drank less alcohol per week, were less likely to have self-exempting beliefs, and were more likely to have self-efficacy beliefs regarding quitting, to believe that smoking had damaged their health, and to report that smoking was never allowed anywhere in their home. In multiple logistic regression analysis, higher education level, having a religious affiliation, and a higher self-efficacy regarding quitting were significantly associated with intention to quit.

**Conclusions:**

Sociodemographic factors, smoking-related beliefs, and smoking restrictions at home were associated with intention to quit smoking among Korean adults.

## INTRODUCTION

Intention to quit smoking has received attention in smoking cessation research. A panel study reported that, with regard to sociodemographic variables, older age, less education, and lower income were associated with absence of intention to quit.^1^ It has also been reported that the social and cognitive factors associated with intention to undergo smoking cessation treatment were old age, nicotine dependency, social encouragement to abstain, and having a positive attitude towards smoking cessation treatment.^2^

In particular, smoking-related beliefs such as self-exempting beliefs and self-efficacy are associated with intention to quit.^3^^–^^5^ When smokers experience a discrepancy between their desire to quit and their continuing smoking behavior, they are likely to experience psychological discomfort, known as cognitive dissonance.^6^ Smokers may hold justifications to reduce cognitive dissonance by minimizing the dangers of smoking or positing that the risks are worth the benefits (called self-exempting beliefs). Although justifying smoking helps smokers reduce dissonance, they do so at the cost of continuing an extremely harmful behavior. Research has shown that justifications are negatively related to intention to quit.^3^^,^^4^^,^^7^ Also, several studies have reported that self-efficacy, ie, the belief in one’s capabilities to organize and execute the courses of action required to manage prospective situations,^8^ is a predictor of intention to quit.^1^^,^^9^

Regarding smoking restrictions, studies reported that smoke-free homes and workplaces are associated with more attempts to quit, reduced cigarette consumption, and lower smoking prevalence.^10^^–^^14^ To our knowledge, however, few studies have explored the relationship between smoking restrictions and intention to quit. Myung et al found that smokers who reported household smoking restrictions were more likely to intend to quit smoking as compared with those who did not.^15^

Few studies have investigated the factors related to intention to quit among smokers in Korea, where the smoking rate in men is the second highest among countries in the Organisation for Economic Co-operation and Development (OECD).^16^ A study reported that the variables which predicted intention to quit in Korean adult smokers were smoking temptation, advantages of smoking, disadvantages of smoking, and self re-evaluation.^17^

The purpose of this study was to examine sociodemographic characteristics, smoking-related beliefs, and smoking restrictions as predictors of intention to quit in Korean adults.

## METHODS

### Sample

We used data from the International Tobacco Control Korea Survey (ITC-Korea), which is an ongoing survey of adult male and female smokers aged 19 years or older who had smoked at least 100 cigarettes in their lifetime and at least once in the past 30 days. The data were collected from November through December 2005 by means of a computer-assisted telephone interviewing using random-digit dialing. The survey protocol was based on the International Tobacco Control Policy Evaluation Project (ITC Project) protocol, partially adapted to Korea. The ITC-Korea cohort was constructed from 16 metropolitan areas and provinces from a random sampling of telephone numbers stratified according to relative population size. Our 94 specially trained interviewers conducted the survey in 2 steps: (1) a 9-minute recruitment attempt among respondents who satisfied the inclusion criteria and (2) a 47-minute questioning session. A total of 1002 (71.5%) of the 1402 eligible respondents gave complete interviews. All respondents gave informed consent, and the study was approved by the institutional review board of the National Cancer Center, Korea and the Research Ethics Board of the University of Waterloo, Canada.

### Measures

#### Intention to quit as the main dependent variable

We measured intention to quit by using the following question: “Are you planning to quit smoking?” The 4 possible answers were “within the next month”, “within the next 6 months”, “sometime in the future, beyond 6 months”, and “not planning to quit”. We used a dichotomous variable in the analysis: any of the first 3 responses was defined as having an intention to quit and the last response was defined as not having an intention to quit.

#### Baseline sociodemographic variables

The baseline sociodemographic variables were age, sex, number of cigarettes smoked per day, marital status, annual income, education level, religious affiliation, and number of alcoholic drinks per week.

#### Smoking-related beliefs

Self-exempting beliefs. Respondents were asked, “Please tell me if you strongly agree, agree, neither agree nor disagree, disagree, or strongly disagree with each of the following statements: 1) the medical evidence that smoking is harmful is exaggerated, 2) you’ve got to die of something, so why not enjoy yourself and smoke, and 3) smoking is no riskier than lots of other things people do”. We categorized the responses into strongly agree/agree or other.

Self-efficacy of quitting. Respondents were asked, “If you decided to give up smoking completely in the next 6 months, how sure are you that you would succeed?”, and were given a choice of 5 answers: not at all sure, somewhat sure, moderately sure, very sure, and extremely sure. We categorized these responses as extremely/very/moderately sure or other.

Degree of difficulty in quitting. Respondents were asked, “How easy or hard would it be for you to quit smoking if you wanted to?”, and were given a choice of 5 answers: very easy, somewhat easy, neither easy nor hard, somewhat hard, and very hard. We categorized their responses as very/somewhat hard or other.

Degree of damage to one’s health from smoking. Respondents were asked, “To what extent, if at all, has smoking damaged your health?”, and were given a choice of 4 answers: not at all, just a little, a fair amount, and a great deal. We categorized their responses as a great deal/a fair amount or other.

#### Smoking restrictions

Smoking at home. Respondents were asked, “Which of the following best describes smoking in your home?”, and were given a choice of 3 answers: smoking is allowed anywhere in my home, smoking is never allowed anywhere in my home, and something in between. The responses were categorized as allowed anywhere/something in between or never allowed anywhere.

Smoking in workplaces. Respondents were asked, “Which of the following best describes the smoking policy where you work?”, and were given a choice of 3 answers: smoking is not allowed in any indoor area, smoking is allowed only in some indoor areas, and smoking is allowed in all indoor areas. The responses were categorized as allowed in all indoor areas/in some indoor areas or not allowed in any indoor area.

Smoking in drinking establishments. Respondents were asked, “Which of the following best describes the rules about smoking in drinking establishments, bars, and pubs where you live?”, and were given a choice of 3 answers: smoking is not allowed in any indoor area, smoking is allowed only in some indoor areas, and no rules or restrictions. The responses were categorized as no rules or restrictions/allowed only in any indoor area or not allowed in any indoor area.

Smoking in restaurants and cafes. Respondents were asked, “Which of the following best describes the rules regarding smoking in restaurants and cafes where you live?”, and were given a choice of 4 answers: smoking is not allowed in any indoor area, smoking is allowed only in some indoor areas, smoking is allowed in all indoor areas, and every café and restaurant has its own rules. The responses were categorized as allowed only in some indoor areas/in all indoor areas/every café and restaurant has its own rules or not allowed in any indoor area.

### Statistical analysis

We used the Pearson chi-square test (for categorical variables) and a 2-sample *t*-test (for continuous variables) to test the significance of baseline differences in sociodemographic characteristics between smokers who had an intention to quit smoking and those who did not. We examined predictors of intention to quit by using univariate analysis. In addition, we performed multiple logistic regression analysis adjusted for all the variables, ie, age, sex, number of cigarettes smoked per day, marital status, annual income, education level, number of alcoholic drinks per week, self-exempting beliefs, self-efficacy of quitting, degree of difficulty in quitting, degree of damage to one’s health from smoking, and smoking restrictions at home, workplaces, drinking establishments, and restaurants and cafes. Additionally, we conducted multiple logistic regression analysis by generation (<40 or ≥40 years) to examine differences between the younger and older generations in independent variables associated with intention to quit. All statistical tests were 2-sided, and a *P* value of 0.05 or less was considered to indicate statistical significance. We used SPSS 12.0K for Windows for the data analyses.

## RESULTS

### Baseline characteristics

Because of missing data, 995 of the 1002 enrolled respondents were included in the final analysis. Of those, 74.9% (*n* = 745) intended to quit smoking. Table 1 shows the differences in baseline characteristics between smokers with and without an intention to quit. As compared with smokers without an intention to quit, those with an intention to quit were younger, smoked fewer cigarettes per day, had a higher annual income, were more educated, were more likely to name a religious affiliation, and drank less alcohol per week. Sex and marital status were not significantly associated with intention to quit.

**Table 1.** Baseline characteristics of the respondents according to intention to quit smoking (*n* = 995)

Baseline characteristics	Smokers without intention to quit (*n* = 250)	Smokers with intention to quit (*n* = 745)	*P*^c^
Age (yr)^a^	46.9 ± 14.1	43.7 ± 13.9	0.002
Sex			0.912
Female	10 (24.4)	31 (75.6)	
Male	240 (25.2)	714 (74.8)	
No. of cigarettes smoked per day^a^	20.4 ± 9.9	18.2 ± 8.6	0.001
Marital status			0.826
Not married	74 (24.7)	226 (75.3)	
Married	176 (25.3)	519 (74.7)	
Annual income (won)^b^			0.035
<30 million	123 (28.5)	308 (71.5)	
≥30 million	108 (22.5)	373 (77.5)	
Education level			<0.001
≤High school	179 (29.8)	422 (70.2)	
≥College	71 (18.0)	323 (82.0)	
Religious affiliation			0.014
No	144 (28.5)	362 (71.5)	
Yes	106 (21.7)	383 (78.3)	
No. of alcoholic drinks/week			0.044
≤2	162 (23.3)	533 (76.7)	
≥3	88 (29.3)	212 (70.7)	

Values = number (%) unless otherwise indicated. ^a^Mean ± SD. ^b^1150 won = $US 1.

^c^Pearson chi-square test for categorical variables and 2-sample *t*-test for continuous variables.

### Univariate analysis

Table 2 shows smoking-related beliefs and smoking restrictions associated with intention to quit. As compared with smokers who did not intend to quit smoking, smokers who intended to quit were significantly less likely to have the 3 investigated self-exempting beliefs. Smokers who were sure that they would succeed if they decided to give up smoking completely in the next 6 months (self-efficacy regarding quitting) were more likely to have an intention to quit smoking (odds ratio [OR], 2.89; 95% CI, 2.15–3.89). Similarly, those who believed that smoking had damaged their health were more likely to intend to quit (2.78, 2.01–3.84). With regard to smoking restrictions, never being allowed to smoke anywhere at home was significantly associated with intention to quit.

**Table 2.** Smoking-related beliefs and smoking-restriction variables associated with having an intention to quit smoking in univariate analysis (*n* = 995)

Variables	OR (95% CI)
**Self-exempting beliefs**	
“The medical evidence that smoking is harmful is exaggerated.”	
Other	1
Strongly agree/agree	0.56 (0.41–0.76)
“You’ve got to die of something, so why not enjoy yourself and smoke.”	
Other	1
Strongly agree/agree	0.50 (0.37–0.67)
“Smoking is no riskier than lots of other things that people do.”	
Other	1
Strongly agree/agree	0.70 (0.52–0.93)
**Self-efficacy regarding quitting**	
“If you decided to give up smoking completely in the next 6 months, how sure are you that you would succeed?” (Self-efficacy regarding quitting smoking)	
Other	1
Extremely/very/moderately sure	2.89 (2.15–3.89)
**Degree of difficulty in quitting**	
“How easy or hard would it be for you to quit smoking if you wanted to?”	
Other	1
Very/somewhat hard	1.13 (0.79–1.61)
**Degree of damage to one’s health from smoking**	
“To what extent, if at all, has smoking damaged your health?”	
Other	1
A great deal/a fair amount	2.78 (2.01–3.84)
**Smoking-restriction variables**	
Smoking at home	
Never allowed anywhere	1
Allowed anywhere/something in between	0.68 (0.49–0.94)
Smoking at work	
Not allowed in any indoor area	1
Allowed in any indoor areas/in some indoor areas	0.74 (0.50–1.10)
Smoking in drinking establishments	
Not allowed in any indoor area	1
No rules or restrictions/allowed only in any indoor area	1.17 (0.62–2.20)
Smoking in restaurants and cafes	
Not allowed in any indoor area	1
Allowed in all indoor areas/only in some indoor areas/every café and ​ restaurant has its own rules	1.00 (0.64–1.64)

### Multiple logistic regression analysis

Table 3 shows associations of baseline characteristics, smoking-related beliefs, and smoking-restriction variables with intention to quit in multiple logistic regression analysis.

**Table 3.** Baseline characteristics, smoking-related beliefs, and smoking-restriction variables associated with intention to quit smoking in multiple logistic regression analysis (*n* = 995)

Variables	OR^a^ (95% CI)		

	All	Younger generation (<40 years) (*n* = 387)	Older generation (≥40 years) (*n* = 608)
Age (continuous variable)	0.99 (0.97–1.00)	n.a.	n.a.
Sex			
Female	1	1	1
Male	0.76 (0.33–1.73)	0.78 (0.34–1.79)	0.51 (0.17–1.55)
No. of cigarettes smoked per day			
<20	1	1	1
≥20	0.74 (0.52–1.06)	0.74 (0.52–1.05)	0.68 (0.43–1.07)
Marital status			
Not married	1	1	1
Married	1.15 (0.77–1.74)	1.02 (0.70–1.48)	1.35 (0.75–2.42)
Annual income (won)^b^			
<30 million	1	1	1
≥30 million	1.06 (0.73–1.53)	1.16 (0.82–1.64)	0.86 (0.54–1.37)
Education level			
≤High school	1	1	1
≥College	1.56 (1.08–2.27)	1.65 (1.15–2.38)	2.11 (1.26–3.53)
Religious affiliation			
No	1	1	1
Yes	1.46 (1.04–2.06)	1.42 (1.01–2.00)	1.34 (0.87–2.04)
No. of alcoholic drinks per week			
≤2	1	1	1
≥3	0.79 (0.55–1.13)	0.79 (0.55–1.13)	0.74 (0.47–1.16)
**Self-exempting beliefs**			
“The medical evidence that smoking is harmful is exaggerated.”			
Other	1	1	1
Strongly agree/agree	0.72 (0.50–1.04)	0.72 (0.50–1.04)	0.69 (0.43–1.10)
“You’ve got to die of something, so why not enjoy yourself and smoke.”			
Other	1	1	1
Strongly agree/agree	0.69 (0.49–1.00)	0.69 (0.48–0.98)	0.55 (0.35–0.87)
“Smoking is no riskier than lots of other things that people do.”			
Other	1	1	1
Strongly agree/agree	0.80 (0.56–1.14)	0.81 (0.57–1.15)	0.86 (0.55–1.34)
**Self-efficacy regarding quitting**			
“If you decided to give up smoking completely in the next 6 months, how sure are you that you would succeed?” (Self-efficacy regarding quitting smoking)			
Other	1	1	1
Extremely/very/moderately sure	3.08 (2.16–4.38)	3.04 (2.14–4.33)	3.23 (2.07–5.03)
**Degree of difficulty in quitting**			
“How easy or hard would it be for you to quit smoking if you wanted to?”			
Other	1	1	1
Somewhat hard/very hard	1.93 (1.24–2.98)	1.92 (1.24–2.97)	2.42 (1.38–4.27)
**Degree of damage to one’s health from smoking**			
“To what extent, if at all, has smoking damaged your health?”			
Other	1	1	1
A great deal/a fair amount	2.69 (1.86–3.90)	2.74 (1.89–3.96)	2.65 (1.67–4.22)
**Smoking-restriction variables**			
Smoking allowance in home			
Never allowed anywhere	1	1	1
Allowed anywhere/something in between	0.91 (0.62–1.33)	0.89 (0.61–1.30)	1.00 (0.61–1.62)
Smoking allowance in workplaces			
Not allowed in any indoor area	1	1	1
Allowed in any indoor areas/in some indoor areas	0.96 (0.61–1.53)	0.93 (0.59–1.47)	1.34 (0.74–2.45)
Smoking allowance in drinking establishments			
Not allowed in any indoor area	1	1	1
No rules or restrictions/allowed only in any indoor area	1.12 (0.52–2.39)	1.21 (0.57–2.57)	1.36 (0.61–3.04)
Smoking allowance in restaurants and cafes			
Not allowed in any indoor area	1	1	1
Allowed in all indoor areas/only in some indoor ​ areas/every café and restaurant has its own rules	1.03 (0.58–1.83)	1.08 (0.61–1.91)	1.27 (0.66–2.44)

^a^Odds ratios were adjusted for all variables above. ^b^1150 won = $US 1.

With regard to sociodemographic characteristics, a higher education level and religious affiliation were associated with intention to quit. In addition, higher self-efficacy regarding quitting was significantly associated with intention to quit (OR, 3.08; 95% CI, 2.16–4.38) in multiple logistic regression analysis, whereas no self-exempting belief was significantly associated with intention to quit. In addition, the perceived difficulty of quitting and damage to one’s health from smoking were significant predictors of intention to quit. No smoking-restriction variable was associated with intention to quit.

In multiple logistic regression analysis by generation (<40 or ≥40 years), the findings were similar to those from multiple logistic regression analysis of all respondents. However, in contrast to the younger generation, having a religious affiliation was not associated with intention to quit in the older generation. In addition, smokers who agreed that “you’ve got to die of something, so why not enjoy yourself and smoke” were less likely to intend to quit among both the younger and older generations.

## DISCUSSION

In Korean adults, intention to quit smoking was associated with sociodemographic characteristics (younger age, lower cigarette consumption, higher annual income, higher education level, having a religious affiliation, and lower alcohol consumption), smoking-related beliefs (fewer self-exempting beliefs, greater self-efficacy regarding quitting, and believing that smoking damaged health), and 1 smoking-restriction variable (never being allowed to smoke anywhere at home). After controlling for all factors used in the univariate analyses, predictors of intention to quit were a higher education level, having a religious affiliation, a higher self-efficacy regarding quitting, believing that quitting smoking is hard, and that smoking had damaged one’s health to a fair or great extent.

Several studies have found that the sociodemographic predictors of intention to quit in adult smokers were higher income, younger age, lower daily cigarette consumption, and being married.^1^^,^^18^^,^^19^ Our findings were consistent with those studies, with the exception of marital status. The ITC Four Country Survey (ITC-4 Survey) reported that women were more likely than men to intend to quit,^1^ but our results showed no sex difference in intention to quit. This may be due to the lower sample size in the Korean versus the ITC-4 survey and to the very low prevalence of smoking among Korean women.

One study reported that education level was not associated with intention to quit, which conflicts with our findings.^20^ In a Dutch study that tested the theory of planned behavior and adjusted for age, sex, marital status, and religious affiliation, Droomers et al concluded that only the direct effect of self-efficacy on behavior contributed to the effect of educational level on quitting smoking.^20^ However, the large (*n* = 8000) ITC-4 Survey, which included the United States, Canada, the United Kingdom, and Australia, reported that low education was significantly associated with the absence of intention to quit, when adjusted for age, sex, amount of smoking, low self-efficacy, country, and income (adjusted OR, 1.40; 95% CI, 1.23–1.60).^1^ A possible reason for these divergent findings is that the respective studies used different operational definitions of intention to quit. The present study and the ITC-4 Survey defined intention to quit as intention to quit within the next 6 months, while Droomers et al defined it as intention to quit within the next month.

Our study showed that smokers with self-exempting beliefs were less likely to intend to quit smoking in univariate analyses, even though those findings were not shown in multiple logistic regression analysis. Previous studies also reported a negative association between self-exempting beliefs and intention to quit.^3^^,^^4^^,^^7^

Smoking-related beliefs such as self-efficacy and perceived damage to health from smoking were significantly associated with intention to quit in both univariate and multiple logistic regression analyses. These findings are consistent with previous reports.^1^^,^^9^

Few studies have explored the relationship between smoking restrictions and intention to quit, although several have reported an association between the presence of smoke-free places and smoking behavior.^10^^–^^14^ It has been reported that smokers living in households where smoking was banned were more likely to be contemplating quitting or preparing to quit^11^ and that smokers who reported having household smoking restrictions were more likely to intend to quit as compared with those who did not have such restrictions.^15^ Similarly, in the present univariate analyses, smokers living in households where smoking was allowed were less likely to intend to quit. Smoking rules in workplaces, drinking establishments, and restaurants and cafes, however, were not associated with intention to quit. This may be attributable to a qualitative difference in smoking restrictions in the home versus the workplace. That is, while a smoking ban in a workplace is usually implemented as part of company policy, regardless of the smoker’s will, a smoking ban at home might be implemented based on family agreement, which means the smoker may have already been thinking of quitting. More studies are needed to confirm the relationship between smoking restrictions and intention to quit.

In multiple logistic regression analysis stratified by generation (<40 or ≥40 years), we observed no obvious differences in the independent variables that were associated with intention to quit. There was a significant negative association between the self-exempting belief “You’ve got to die of something, so why not enjoy yourself and smoke” and intention to quit in both generations; the association was only marginally negative when the generations were combined. Even though we found no significant association between other self-exempting beliefs and intention to quit, a negative association might be observed in a larger study, because the upper limits were close to 1 in our multiple logistic analysis.

Our study had several limitations. First, because of the nature of our cross-sectional data, we could not observe the relationship between intentions or attempts to quit and actual quitting; prospective research is needed. Second, because the number of adult female smokers enrolled in the study was small, the statistical power of the data on women was too low to investigate the present variables. The smoking rate among adult women in Korea is extremely low (<5%) as compared with that of men.^16^ Further studies with a larger number of women smokers are required.

In summary, this nationwide cross-sectional survey within strata defined by geographic region and community size showed that sociodemographic factors, smoking-related beliefs, and smoking restrictions at home are associated with intention to quit smoking among Korean adult smokers.
